# A higher preconceptional paternal body mass index influences fertilization rate and preimplantation embryo development

**DOI:** 10.1111/andr.13128

**Published:** 2021-11-25

**Authors:** Jeffrey Hoek, Sam Schoenmakers, Linette van Duijn, Sten P. Willemsen, Eva S. van Marion, Joop S. E. Laven, Esther B. Baart, Régine P. M. Steegers‐Theunissen

**Affiliations:** ^1^ Department of Obstetrics and Gynecology Erasmus MC University Medical Center Rotterdam The Netherlands; ^2^ Department of Biostatistics Erasmus MC University Medical Center Rotterdam The Netherlands; ^3^ Division of Reproductive Endocrinology and Infertility Department of Obstetrics and Gynecology Erasmus University Medical Center Rotterdam The Netherlands

**Keywords:** assisted reproductive techniques/ICSI, body mass index, embryonic development, male, obesity, spermatozoa

## Abstract

**Background:**

Obesity is a worldwide problem affecting the health of millions of people throughout the life course. Studies reveal that obesity impairs sperm parameters and epigenetics, potentially influencing embryonic development.

**Objective:**

To investigate the association between preconceptional paternal body mass index (BMI) and embryo morphokinetics using a time‐lapse incubator and in vitro fertilization (IVF) and intracytoplasmic sperm injection (ICSI) outcomes.

**Materials and methods:**

Participants were recruited from a tertiary hospital in this prospective periconceptional cohort study. A total of 211 men were included: 86 with normal weight (BMI < 25.0), 94 overweight (BMI 25–29.9), and 41 obese (BMI ≥ 30). These men were part of a couple that underwent IVF/ICSI treatment with ejaculated sperm after which 757 embryos were cultured in a time‐lapse incubator. The main outcome parameters consisted of fertilization rate, embryo developmental morphokinetics, embryo quality assessed by a time‐lapse prediction algorithm (KIDScore), and live birth rate.

**Results:**

A higher paternal BMI was associated with faster development of the preimplantation embryo, especially during the first cleavage divisions (*t*2: −0.11 h (*p = *0.05) and *t*3: −0.19 h (*p = *0.01)). Embryo quality using the KIDScore was not altered. The linear regression analysis, after adjustment for confounders (paternal age, ethnicity, smoking, alcohol use, education, total motile sperm count, and maternal age and BMI), showed an inverse association between paternal BMI and fertilization rate (effect estimate: −0.01 (*p* = 0.002)), but not with the live birth rate.

**Discussion and conclusion:**

Our data demonstrate that a higher preconceptional paternal BMI is associated with a reduced fertilization rate in IVF/ICSI treatment. Our findings underline the importance of a healthy paternal weight during the preconception period.

## 1 INTRODUCTION

Overweight and obesity are worldwide problems affecting the health of millions of people throughout the life course.^1^ The pathophysiologic origin of obesity is complex and results from the interplay between inadequate dietary intake, limited exercise, and genetic predisposition.^2^ The prevalence of obesity is also high in the reproductive population, with estimates up to 50%. The influence of obesity on reproductive health is widely studied in women, showing lower oocyte and embryo quality, a longer time to pregnancy, and increased risks of congenital malformations, miscarriages,^3^, ^4^, ^5^ preeclampsia, preterm birth, and fetal death.^6^ However, the negative effects of obesity in men in the reproductive period are largely neglected and understudied. With the global burden of the male obesity,^7^ we hypothesize that periconceptional paternal obesity also affects reproductive outcome.

Studies reveal that obesity impairs sperm concentration, motility, and sperm deoxyribonucleic acid (DNA) quality.^8^, ^9^ Obesity is characterized by a systemic and chronic inflammatory state, with adipocytes continuously releasing inflammatory factors and thereby inducing a pro‐inflammatory state and excessive oxidative stress that increases sperm DNA damage.^10^ The increased exposure to reactive oxidative species due to excessive oxidative stress is also associated with changes in DNA methylation patterns and chromatin constitution during spermatogenesis, with a potential impact on a paternal epigenetic contribution on subsequent embryogenesis.^11^, ^12^, ^13^ The influence of paternal obesity on the preimplantation embryo development has scarcely been studied in human with conflicting results.^14^

In vitro preimplantation embryo development is of interest since it provides a unique insight into the direct impact of paternal factors through sperm, undisturbed by the effects of the maternal in vivo uterine environment. Since obesity itself is associated with sperm quality parameters and pregnancy chance, we hypothesize that preconception paternal weight is also associated with preimplantation embryo development and fertility treatment outcomes. Maternal health also remains of interest since primordial germ cells up to the oocyte maturation period are exposed to the intrinsic maternal environment. In addition, maternal obesity is known to be associated with decreased oocyte quality.^15^

Preimplantation embryo development can be studied using time‐lapse embryo culture, which uses incubators with a built‐in microscope designed for automated time‐lapse embryo assessment by acquiring images. This provides a controlled culture environment and captures comprehensive information on embryo development without the need for handling or disturbing the developing embryo. The use of timing of embryo developmental events, also referred to as embryo morphokinetic parameters, has added another dimension to current traditional morphology classification scores.

Because of the epidemic burden of obesity, which also involves men of reproductive age, the main aim of this study is to investigate associations between preconception paternal obesity and developmental morphokinetics of preimplantation embryos and specific in vitro fertilization–intracytoplasmic sperm injection (IVF–ICSI) treatment outcomes.

## 2 MATERIALS AND METHODS

### 2.1 Ethical approval

This study was conducted according to the guidelines laid down in the Declaration of Helsinki and all procedures involving patients were approved by the Medical Ethical Institutional Review Board of the Erasmus, University Medical Centre, Rotterdam, the Netherlands. Written informed consent was obtained from all participants at enrolment.

### 2.2 Study design, population, and patient inclusion

Couples were enrolled in the prospective virtual embryoscope study, which is embedded in the Rotterdam Periconception Cohort (predict study).^16^ The predict study is an ongoing prospective tertiary hospital‐based cohort embedded in the outpatient clinic of the Department of Obstetrics and Gynecology of the Erasmus MC, University Medical Center Rotterdam, the Netherlands. Patients were eligible for inclusion in the virtual embryoscope study if they were subfertile, based on either male of female factor subfertility, with an indication for IVF treatment, with or without ICSI. Furthermore, participants needed to be at least 18 years of age and had to read and understand good Dutch. Criteria for exclusion consisted of oocyte donation and not able to understand the Dutch language. Couples were included between May 2017 until December 2019 at the Erasmus MC, University Medical Centre, Rotterdam, the Netherlands.

### 2.3 In vitro fertilization procedures

Ovarian stimulation, oocyte retrieval, the IVF/ICSI procedures, and assessment of embryo morphology were performed as described previously.^17^, ^18^ After the ICSI treatment, injected oocytes were directly placed in the EmbryoScope™ in Sage one‐step culture medium (Origio/Cooper Surgical™, Denmark) at 36.8°C, 7% oxygen and 5% carbon dioxide. Embryos after IVF treatment were cultured in the EmbryoScope™ after the appearance of both pronuclei. Embryo images were automatically recorded in seven focal planes (15 μm intervals, 1280 × 1024 pixels, 3 pixels/μm, monochrome CCD camera, single red LED 635 nm duration < 0.1 s/image, total light exposure time < 50 s/day/embryo) every 10 min until embryo day 3.

Embryo evaluation and selection for transfer was carried out on day 3 after oocyte retrieval, based on developmental stage and morphology as assessed on the last time‐lapse image acquired 66–68 h after fertilization. Embryo selection for transfer was not aided by time‐lapse information. Embryos were ranked according to number of blastomeres, fragmentation, size equality, and signs of early compaction. Top‐ranked embryos consisted of eight equally sized blastomeres with little (< 10%) or no fragmentation.

### 2.4 Study parameters

Standardized anthropometric measurements for both men and women were carried out, including paternal and maternal height with 0.1 cm accuracy and weight with 0.1 kg accuracy (anthropometric rod and weighing scale; SECA, Hamburg, Germany). Participants completed a self‐administered questionnaire covering details on age, ethnicity, educational level and preconceptional use of alcohol, cigarettes, and folic acid supplements. All data were verified at study entry and anthropometrics were measured by a researcher.

Before processing for IVF or ICSI, quality of the semen sample was routinely assessed. Total motile sperm count (TMSC), which is obtained by multiplying the volume of the ejaculate in milliliters by the sperm concentration and the proportion of A (fast forward progressive) and B (slow progressive) motile sperms divided by 100%. All semen parameters were assessed according to the 2010 WHO guideline laboratory manual for the examination and processing of human semen.^19^

Time‐lapse parameters were annotated manually according to the definitions and guidelines of the ESHRE consensus for dynamic monitoring of the human preimplantation development.^20^ All freshly transferred and cryopreserved embryos were individually annotated for the following morphokinetic parameters: tPNf, *t*2, *t*3, *t*4, *t*5, *t*6, *t*7, and *t*8. tPNf was defined as the first frame in which both pronuclei had faded. The timing of reaching the 2‐, 3‐, 4‐, 5‐, 6‐, 7‐, and 8‐cell stage were defined as *t*2, *t*3, *t*4, *t*5, *t*6, *t*7, and *t*8, respectively. These parameters were used by the Vitrolife® embryo‐viewer software to calculate intervals between cleavages, as well as to assign each embryo a Known Implantation Data (KID) Score.^21^ This is a generally applicable embryo deselection tool based on six parameters, of which the lowest score ( = 1) is reported to correspond with a low chance of implantation, whereas the highest score ( = 5) corresponds with a high chance of implantation.^21^ Internal validation of inter‐observer reproducibility of annotations between team members demonstrated extremely close agreement for the timings of the pronuclear stage until the 5‐cell cleavage divisions (intraclass correlation coefficient, ICC > 0.95). A moderate agreement was found for cleavage divisions between the 6‐ and 8‐cell stage (ICC 0.23–0.40).

Fertility treatment outcomes were retrieved from medical records. Fertilization rate was calculated by dividing the number of fertilized oocytes by the total number of metaphase II oocytes retrieved. The embryo usage rate was calculated by dividing the total number of usable embryos per patient, that is, all embryos transferred or cryopreserved, by the number of fertilized oocytes. Additionally, positive pregnancy test, visible fetal heartbeat around 12 weeks of gestation, and livebirth data were collected after fresh transfer.

### 2.5 Statistical analysis

Baseline characteristics of men are depicted as median or number with the corresponding interquartile range (IQR) or percentage. All analyses were performed using SPSS package 25.0 (IBM SPSS Statistics, Armonk, NY). R (R: A language and Environment for Statistical Computing, version 3.1.3, 2015 for Windows, R Core Team, Vienna, Austria) was used to perform analysis on the KIDScore using the proportional odds model.

Baseline data were tested for the assumption of normality. If continuous data did not fulfill the assumption of normality, a Kruskal–Wallis test was performed and estimates are reported as medians and IQR. Categorical baseline data were analyzed using a Chi‐square test/Fishers exact test.

All analyses were performed with body mass index (BMI) as continuous variable. Analyses on the developmental time points of reaching the different cell stages were performed on transferred and cryopreserved embryos. Linear mixed models were applied to study the association between paternal BMI and preimplantation developmental timings. To study associations between paternal BMI and the KIDScore, we used a proportional odds model, which is a model for ordinal outcomes like the KIDScore, using the ordinal package in R (Rune Haubo B Christensen). Random subject effects are used in the proportional odds model to account for the clustering of multiple embryos of one couple.

The dichotomous treatment outcomes, like positive pregnancy test, fetal heartbeat and live birth, were analyzed using logistic regression and the continuous treatment outcomes, like fertilization rate and embryo usage rate, were analyzed using standard linear regression.

For linear mixed models, proportional odds models, logistic regression, and linear regression, we used different models to account for confounding factors. Confounders were selected based on the available literature and significant factors from the correlation matrix of our study population. In the crude model, we did not adjust for potential confounders and in model 1 we additionally adjust for potential paternal confounders (TMSC, age, ethnicity, smoking, alcohol use, and education) and maternal confounders (BMI, age, smoking, alcohol use, and education) and fertilization type. *p*‐Values < 0.05 were considered statistically significant.

## 3 RESULTS

### 3.1 Baseline

**FIGURE 1:**
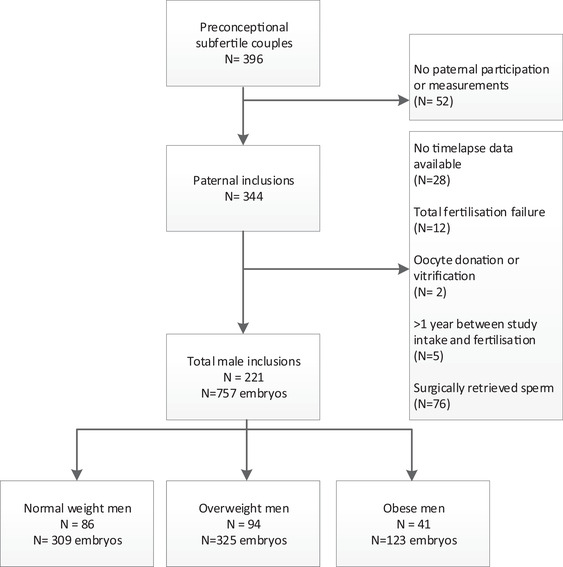
Flowchart of the inclusions and exclusions of the study population

After inclusion of a total of 396 preconceptional subfertile couples, patients were excluded because of no paternal participation (*n* = 52), no available time‐lapse data (*n* = 28), total fertilization failure (*n* = 12), oocyte donation/vitrification (*n* = 2), more than 1 year between study intake and fertilization (*n* = 5) and use of surgically retrieved sperm (*n* = 76). In total, 221 (*n* = 757 embryos) men were included of which 86 (*n* = 309 embryos) were of normal weight, 94 (*n* = 325 embryos) were overweight, and 41 (*n* = 123 embryos) were obese (Figure 1). The median number of cryopreserved and transferred embryos per cycle was 4 (IQR 2–7).

BMI of men with total fertilization failure was not significantly different from normal fertilization (28.4 kg/m^2^ and 26.7 kg/m^2^ (*p* = 0.47)). Also, after logistic regression analysis we did not find an association between paternal BMI and total fertilization failure (*p* = 0.20).

At baseline, we found no significant differences between normal weight, overweight, and obese men regarding the indication of the subfertility treatment (male factor in 33.7%, 36.2%, and 26.8% respectively (resp.) (*p* = 0.17)), or the ovarian stimulation protocol used by the female partner (GnRH‐antagonist co‐treatment in 76.1%, 81.9%, 65.9% resp. (*p* = 0.47)) (Table 1). Differences were observed between normal weight, overweight, and obese men regarding age (34.5, 35.4, and 36.6 years resp. (*p* = 0.01)), geographic origin (88.4%, 77.7%, and 73.2% Dutch origin resp. (*p* = 0.01)) and alcohol use (62.8%, 71.3%, and 48.8% resp. (*p* = 0.01)) (Table S1). Significantly more men were highly educated in the normal weight group (54.7%) compared to the overweight (35.5%) and obese group (22.0%) (*p* = 0.01).

**TABLE 1:** Subfertility parameters of men and women in the study population of the virtual embryoscope study

	**Normal weight men*N* = 86**	**Overweight men*N* = 94**	**Obese men*N* = 41**	***p*‐Value**
**Indications for subfertility treatment: *n* (%)**				
Male factor	29 (33.7%)	34 (36.2%)	11 (26.8%)	0.17
Combined male‐female	10 (11.6%)	18 (19.6%)	14 (34.1%)	
Female factor	32 (37.2%)	33 (35.1%)	10 (24.4%)	
Unexplained	15 (17.4%)	9 (9.6%)	6 (14.6%)	
**Male factors**: ***n* (%)**	***N* = 39 (29 males and 10 combined females–males)**	***N* = 52 (34 males and 18 combined females–males)**	***N* = 25 (11 males and 14 combined females–males)**	0.35
OA(T)	38	49	25	
An‐ and retrograde ejaculation	1	3	0	
**Female factors**: ***n* (%)**	***N* = 42 (32 females and 10 combined females–males)**	***N* = 51 (33 females and 18 combined females–males)**	***N* = 24 (10 females and 14 combined females–males)**	0.51
Tuba factor	7	10	6	
Ovulation disorder	18	23	8	
Endometriosis	11	13	9	
Others	6	5	1	
**Oocytes aspired**	9 [5–14]	8 [5.5–13]	8 [5–11.5]	0.59
**Ovarian stimulation: *n* (%)**				0.47
GnRH‐Agonist	22 (25.6%)	17 (18.1%)	14 (34.1%)	
GnRH‐Antagonist	64 (76.1%)	77 (81.9%)	27 (65.9%)	
**Fertilization type** ***n* (%)**				0.35
IVF	46 (53.5%)	42 (44.7%)	14 (34.1%)	
ICSI	40 (46.5%)	52 (55.3%)	27 (65.9%)	
**TMSC (×10^6^), IQR**	25.8 [2.7–110.3]	12.1 [0.2–85.8]	8.0 [0.6–55.4]	0.09

Abbreviations: GnRH, gonadotropin‐releasing hormone; ICSI, intracytoplasmic sperm injection; IVF, in vitro fertilization; IQR, interquartile range; OAT; oligoasthenoteratozoospermia, TMSC, total motile sperm count.

### 3.2 Total motile sperm count as a sperm quality parameter

In normal weight men, TMSC was 25.8 × 10^6^ IQR (2.7–110.3), whereas this was lower in overweight men (12.1 × 10^6^ IQR (0.2–85.8)) and obese men (8.0 × 10^6^ IQR (0.6–55.4)), however not significantly (*p* = 0.09) (Table 1). In linear regression analysis, paternal BMI was negatively predictive for TMSC (beta: −2.48 million (*p* = 0.11)), however not significantly (Table 2).

**TABLE 2:** Periconceptional paternal body mass index (BMI) and associations with sperm quality, IVF/ICSI treatment outcomes and pregnancy outcomes

	**Crude**			**Model 1**		
**Table 2a**	**Beta (SE)**		***p*‐Value**	**Beta (SE)**		***p*‐Value**
TMSC^a^ *linear regression*	−2.61 (1.50)		0.08	−2.48 (1.53)		0.11
KIDScore ordinal model	−0.01 (0.02)		0.64	−0.01 (0.02)		0.62
Fertilization rate linear regression	−**0.01 (0.003)**		**0.001**	−**0.01 (0.004)**		**0.002**
Embryo usage rate linear regression	−0.001 (0.004)		0.99	−0.001 (0.004)		0.84
Table 2b	Beta	OR	*p*‐Value	Beta	OR	*p*‐Value
Positive pregnancy test logistic regression	0.02	1.02	0.57	0.03	1.03	0.49
Fetal heartbeat logistic regression	0.01	1.01	0.80	0.03	1.03	0.51
Live birth logistic regression	−0.01	0.99	0.84	0.01	1.01	0.82

*Notes*:

Model 1: model 1 + paternal adjustments (total motile sperm count, age, ethnicity, smoking, alcohol use and education) and maternal adjustments (BMI, age, ethnicity, smoking, alcohol use, education).

^a^ Only adjustments for paternal confounders in model 1.

Abbreviations: IVF/ICSI, in vitro fertilization/intracytoplasmic sperm injection; KIDScore, known implantation data score; OR, odds ratio; se, standard error; TMSC, total motile sperm count.

### 3.3 Embryo morphokinetic parameters

The crude results of the linear mixed models for paternal BMI as continuous variable showed that the effect estimates of all morphokinetic time points were negative, indicating that for every increase in BMI point embryos develop faster from *t*2 (−0.13 h (95% confidence interval (CI) (−0.24 to −0.03)), till *t*7 (−0.18 h (95% CI (−0.37 to 0.01)) and *t*8 (−0.15 h (95% CI (−0.34 to 0.05)) (Table 3). After correcting for maternal and paternal confounders (model 1) these negative associations remain, but are only significantly in the first cleavage divisions *t*2 (−0.11 h (95% CI (−0.21 to 0.001)) and *t*3 (−0.19 h (95% CI (−0.33 to −0.04)). Subgroup analyses in only the IVF or IVF–ICSI patients do not show any significant differences (data not shown).

**TABLE 3:** Results of the linear mixed model with periconceptional paternal body mass index (BMI) as continuous variable and morphokinetic parameters of the preimplantation embryo

**Morphokinetic parameters**	**Crude**		**Model 1**	
	**Beta[95% CI](h)**	***p*‐Value**	**Beta[95% CI](h)**	***p*‐Value**
**tPNa**	−0.01 [−0.12 to 0.09]	0.81	−0.01 [−0.08 to 0.07]	0.86
**tPNf**	−**0.10** **[**−**0.19 to** −**0.01]**	**0.048**	−0.07 [−0.17 to 0.03]	0.15
**tPNf – tPNa**	−0.06 [−0.17 to 0.06]	0.32	−0.04 [−0.15 to 0.07]	0.46
**t2**	−**0.13** **[**−**0.24 to** −**0.03]**	**0.02**	−0.11 [−0.21 to 0.001]	0.05
**t3**	−**0.20** **[**−**0.34 to** −**0.06]**	**0.01**	−**0.19** **[**−**0.33 to** −**0.04]**	**0.01**
**t4**	−0.13 [−0.28 to 0.01]	0.07	−0.10 [−0.25 to 0.05]	0.17
**t5**	−0.14 [−0.32 to 0.05]	0.15	−0.12 [−0.32 to 0.08]	0.23
**t6**	−0.14 [−0.33 to 0.04]	0.12	−0.14 [−0.34 to 0.05]	0.14
**t7**	−0.18 [−0.37 to 0.01]	0.07	−0.15 [−0.36 to 0.06]	0.15
**t8**	−0.15 [−0.34 to 0.05]	0.14	−0.11 [−0.33 to 0.10]	0.29

*Notes*: Model 1: crude model + paternal adjustments (age, ethnicity, smoking, alcohol use and education) and maternal adjustments (BMI, age, ethnicity, smoking, alcohol use, education and conception method).

Abbreviations: 95% CI, 95% confidence interval; t, time‐point; tPNa, timing of pronuclear appearance; tPNf, timing of pronuclear fading.

### 3.4 Embryo quality and treatment outcomes

Embryo morphokinetic quality and implantation potential was assessed using the KIDScore. In the proportional odds model, paternal BMI was not associated with the KIDScore (beta: −0.01 (*p* = 0.64)), which remained the same after adjustment for confounders (beta: −0.01 (*p* = 0.62)) (Table 2).

In normal weight men of the total study group, the fertilization rate was 0.88, whereas the rate decreased significantly to 0.81 and 0.76 in the overweight and obese groups. A comparable, but not significant decrease in the fertilization rate was also observed after both IVF and ICSI treatment (Table S2). In linear regression analysis, paternal BMI was negatively associated with fertilization rate (beta −0.01 (*p* = 0.001)), meaning that with every increase in paternal BMI point, the fertilization rate decreased 1%. After adjustment for both maternal and paternal confounders, the negative association remained significant (beta −0.01 (*p* = 0.002)) (Table 2). Results are comparable when also including men from couples that were initially excluded due to total fertilization failure (beta −0.01 (*p* = 0.005)). The embryo usage rate was not associated with paternal BMI after adjustment for confounders (beta −0.001 (*p* = 0.84). In logistic regression (model 1), paternal BMI was not associated with a positive pregnancy test (OR = 1.03 (*p* = 0.49)), fetal heartbeat (OR = 1.03 (*p* = 0.51)) and live birth (OR = 1.01 (*p* = 0.82)).

## 4 DISCUSSION AND CONCLUSION

### 4.1 Main findings

In this study, we show that a higher paternal BMI is associated with a reduced fertilization rate and faster development of the preimplantation embryo, with stronger effects on the first cleavage divisions as compared to the 6‐ to 8‐cell stage. Paternal BMI was not associated with overall morphokinetic embryonic quality of the day 3 embryo, as determined by the KIDScore embryo evaluation algorithm, the embryo usage rate, embryo implantation, and live birth rate.

### 4.2 Strengths and limitations

Strength of this study is the use of a standardized method to determine BMI by the same two researchers over the complete study period, instead of relying on self‐reported data. We adhered to the WHO definitions of BMI categories to make comparison between studies more convenient. A statistical strength is the fact that we controlled paternal effects for important maternal clinical variables such as BMI and age, allowing us to present paternal effects independent of the maternal effects. In this light, the preimplantation embryo development in vitro allows studying early developmental factors outside of the maternal body, allowing contribution and identification of paternal as well as maternal factors from both parental gametes. The uterine environment makes identification of paternal factors impossible, since factors of the uterine environment could diminish paternal effects, while following early development and implantation in utero is still technically impossible. It remains however important to also take the influence of determinants of maternal health into consideration. Female germ cells enter and undergo the first part of meiosis during the fetal development, and resulting oocytes are then exposed to the intrinsic maternal environment, determined by multiple biologic pathways and exposures for many years before meiotic resumption and ovulation. For example, maternal obesity is associated with excessive chronic oxidative stress which can also lead to decreased oocyte quality as evidenced by lower rates of normally fertilized oocytes and decreased ongoing pregnancy rates.^15^, ^22^

A limitation is that our study was conducted in a time period that culture until day 3 was routine clinical practice in most hospitals, as well as in ours. Therefore, future research should also investigate the associations between periconceptional parental nutrition and lifestyle factors and the outcome after embryo culture until day 5, that is, blastocyst quality. Since the study is an observational cohort, we adjusted for potential maternal and paternal confounders, however, residual confounding cannot be fully excluded. We also did not correct for other potential important confounders, such as stress and medication. A limitation of our study remains a relatively low number of patients in the different subgroups, potentially missing the power to investigate birth outcomes. No previous study investigated the effect of paternal obesity and morphokinetic parameters, therefore a power analysis was not possible beforehand. The posthoc sample size calculation using an α‐level of 0.05 and power of 80%, revealed that our study did include the 190 participants needed to accurately show significance. Other differences also exist between the groups, such as the cause of infertility. Since our study population is too small to correct or stratify for these factors, future larger studies should incorporate these factors in sample size calculation and analyses. Although male obesity is linked to adverse reproductive outcome, it is unknown which specific conditions, such as intrinsic excessive chronic oxidative stress exposure affects spermatozoa, or is a mediator in the causation. Our study did not investigate if different levels of reactive oxygen species (ROS) in semen of men with different weight classes that could lead to oxidative stress in spermatozoa. To further validate our findings we recommend to add the measurement of oxidative stress in seminal plasma in future studies on the same topic.

### 4.3 Interpretation

Our results regarding the impact of BMI on embryo morphokinetics are only supported by previous studies in obese women. These showed a delay in the first cleavage divisions of embryos.^23^, ^24^ In addition, Leary et al. showed that embryos from women with overweight and obesity develop faster into a blastocyst. In this study, alterations in glucose and pyruvate metabolism of the embryo were suggested as an underlying cause, as embryo metabolism is determined by the oocyte and the oocyte may directly inherit such impairments from the maternal environment.^25^ Before fertilization is carried out, sperm are selected by density gradient centrifugation and washing, removing the seminal fluid. The influence on the embryo development of any nutrients present in seminal fluid is therefore limited.

We show negative associations between paternal body weight and fertilization rate. Reports in the literature are conflicting: studies where less than 300 participants were included, reported no association,^26^, ^27^ whereas studies with over 600 participants showed negative associations.^28^ While we show no association between paternal BMI and live birth rate, a recent meta‐analysis showed that paternal obesity is linked to a decreased live birth rate (OR 0.88).^29^ The seven included studies combined included more than 13.000 IVF–ICSI cycles. Importantly, each individual study (ranging from 170 cycles to 25,000 cycles) found non‐significant results, indicating a power problem. It is likely that this is also the case in our study with 221 IVF–ICSI cycles.

Obesity has been shown to negatively influence sperm quality and sperm DNA damage. Different underlying mechanisms, such as (epi)genetic, endocrinological, and environmental effects, are described in the literature linking obesity to sperm quality.^28^ Obesity is strongly linked to decreased sperm count via hyperinsulinemia, increased scrotal temperature, and increased oxidative stress.^30^ The increased oxidative stress is caused by ROS, which are produced as a byproduct in the process of aerobic metabolism necessary for normal physiological function. Obesity is characterized by chronic exposure of tissues to excessive oxidative stress, which can result in DNA‐damage (single‐ and double‐strand breaks and chromosomal rearrangements). Furthermore, obesity is also associated with an increased sperm DNA fragmentation index and leads to decreased mitochondrial function in sperm necessary for seminal propulsion with resulting impaired motility.^31^

Our results regarding early embryo morphokinetics are most likely explained by alterations in epigenetics, such as DNA methylation and/or chromatin constitution, since they are thought to affect cleavage divisions up to the 4‐cell stage.^32^ Epigenetic changes present on the DNA in sperm of essential genes related to growth and development, can mediate the negative effects reported here for embryos of obese men. Obesity‐related epigenetic changes in sperm are reported in the current literature. Significant alterations in DNA methylation levels of imprinted genes were observed in sperm of obese men compared to normal weight men. Significantly, lower DNA methylation levels were observed on Maternally Expressed Gene 3 (MEG3) and Necdin (NDN), but higher levels on the H19 gene.^33^ A recent study shows that paternal aging can impact on the fertilization rate and day 5 embryo quality and that is preceded by deregulated methylation at imprinted genes in sperm.^34^ Interestingly, the methylation status of sperm is dynamic and under environmental pressure. More than 1500 unique genes had altered sperm DNA methylation profiles in six men undergoing Roux‐en‐Y gastric bypass surgery already 1 week after the surgery, which remained until at least 1 year after surgery.^35^

It remains unknown which factors cause obesity related sperm DNA‐methylation differences, however some hypotheses are proposed in the current literature. Several studies have demonstrated that the sperm epigenome is responsive to dietary factors and that negative and positive influences are transferred to future generations.^36^, ^37^, ^38^ Obesity in general is strongly associated with elevated estrogen levels, both in women and men. Results from animal studies suggest that increased exposure to estrogen, by increased activity of aromatase present in fat tissue, may lead to abnormal methylation patterns in sperm cells providing a possible mechanism how body fat mass can impact DNA methylation.^30^, ^39^

Recently, a novel potential epigenetic mechanism was identified.^40^, ^41^ Sperm cells carry different types of ribonucleic acid (RNA) and also the epididymal epithelium produces exosome vesicles, which are able to transfer RNA molecules to the passing sperm cells.^42^ In mice, such RNAs have been shown to be critical for fertilization and pre‐ and post‐implantation embryo development.^43^, ^44^ A study in human sperm cells identified the level of expression of a large number of these human sperm RNAs to be responsive to BMI.^41^

From these data, we hypothesize that obesity‐related molecular mechanisms, hormonal imbalances, diet, and other obesity‐related factors can be involved in the causation of (epigenetic) changes in the sperm quality of obese men. The exact underlying pathophysiologic mechanism in our study remains to be elucidated and future research should focus on investigating the role of underlying pathophysiological mechanisms.

We do not show any significant effect of paternal BMI on the pregnancy rate, fetal heartbeat at 12 weeks and live birth rate. This can be explained by the IVF–ICSI procedure itself, either the ovarian stimulation, culture medium or the fact that with ICSI the embryologist selects the sperm cell, which are additional factors influencing pregnancy and live birth rate and could overrule the epigenetic effects of paternal obesity on sperm.

## 5 CONCLUSION

In this study, we show that paternal body weight has a strong negative association with fertilization rate and embryos that develop faster especially in the first cleavage divisions. We found no associations between paternal body weight and pregnancy chance and live birth rate. Explanations for our findings might be the induced alterations in sperm quality, DNA damage, and epigenetic programming caused by chronic exposure to excessive oxidative stress and altered glucose and estrogen levels. Our results demonstrate a paternal impact on the pre‐implantation embryo development with potential consequences for the post‐implantation embryo. Therefore, more research has to be done to investigate if there is a direct impact of paternal obesity on the reproductive outcomes through mechanisms such as excessive intrinsic oxidative stress. In general, it remains important to advise overweight and obese men to achieve a healthy nutritional state and lose weight prior to treatment to optimize the outcome of a time intensive and expensive fertility treatment.

## CONFLICT OF INTEREST

The authors declare no conflict of interest.

## AUTHOR CONTRIBUTIONS

Régine P. M. Steegers‐Theunissen and Esther B. Baart initiated the research question and supervised all aspects of the study. Joop S. E. Laven and Esther B. Baart were responsible for IVF patients. Jeffrey Hoek, Linette van Duijn, and Eva S. van Marion contributed to data acquisition.

Régine P. M. Steegers‐Theunissen supervised the statistical procedures of the article. Jeffrey Hoek, Linette van Duijn, MR and Sam Schoenmakers wrote the first version of the article. All authors contributed to the writing and the critical revisions of the article and all authors approved and authorized the final version.

## Supporting information

SUPPORTING INFORMATION

SUPPORTING INFORMATION

## References

[1] Ng M , Fleming T , Robinson M , et al. Global, regional, and national prevalence of overweight and obesity in children and adults during 1980–2013: a systematic analysis for the Global Burden of Disease Study 2013. Lancet. 2014;384(9945):766‐781. 24880830 10.1016/S0140-6736(14)60460-8 PMC4624264

[2] Marti A , Martinez‐Gonzalez MA , Martinez JA . Interaction between genes and lifestyle factors on obesity. Proc Nutr Soc. 2008;67(1):1‐8. 18234126 10.1017/S002966510800596X

[3] Lashen H , Fear K , Sturdee DW . Obesity is associated with increased risk of first trimester and recurrent miscarriage: matched case–control study. Hum Reprod. 2004;19(7):1644‐1646. 15142995 10.1093/humrep/deh277

[4] Loveland JB , McClamrock HD , Malinow AM , Sharara FI . Increased body mass index has a deleterious effect on in vitro fertilization outcome. J Assist Reprod Genet. 2001;18(7):382‐386. 11499322 10.1023/A:1016622506479 PMC3455823

[5] van der Steeg JW , Steures P , Eijkemans MJ , et al. Obesity affects spontaneous pregnancy chances in subfertile, ovulatory women. Hum Reprod. 2008;23(2):324‐328. 18077317 10.1093/humrep/dem371

[6] Marchi J , Berg M , Dencker A , Olander EK , Begley C . Risks associated with obesity in pregnancy, for the mother and baby: a systematic review of reviews. Obes Rev. 2015;16(8):621‐638. 26016557 10.1111/obr.12288

[7] Kim KB , Shin YA . Males with obesity and overweight. J Obes Metab Syndr. 2020;29(1):18‐25. 32146733 10.7570/jomes20008 PMC7117999

[8] Campbell JM , Lane M , Owens JA , Bakos HW . Paternal obesity negatively affects male fertility and assisted reproduction outcomes: a systematic review and meta‐analysis. Reprod Biomed Online. 2015;31(5):593‐604. 26380863 10.1016/j.rbmo.2015.07.012

[9] Sermondade N , Faure C , Fezeu L , et al. BMI in relation to sperm count: an updated systematic review and collaborative meta‐analysis. Hum Reprod Update. 2013;19(3):221‐231. 23242914 10.1093/humupd/dms050 PMC3621293

[10] Agarwal A , Saleh RA , Bedaiwy MA . Role of reactive oxygen species in the pathophysiology of human reproduction. Fertil Steril. 2003;79(4):829‐843. 12749418 10.1016/s0015-0282(02)04948-8

[11] Rajendran R , Garva R , Krstic‐Demonacos M , Demonacos C . Sirtuins: molecular traffic lights in the crossroad of oxidative stress, chromatin remodeling, and transcription. J Biomed Biotechnol. 2011;2011:368276. 21912480 10.1155/2011/368276 PMC3168296

[12] Richardson ME , Bleiziffer A , Tuttelmann F , Gromoll J , Wilkinson MF . Epigenetic regulation of the RHOX homeobox gene cluster and its association with human male infertility. Hum Mol Genet. 2014;23(1):12‐23. 23943794 10.1093/hmg/ddt392 PMC3857941

[13] Wu C , Ding X , Li H , Zhu C , Xiong C . Genome‐wide promoter methylation profile of human testis and epididymis: identified from cell‐free seminal DNA. BMC Genomics. 2013;14:288. 23622456 10.1186/1471-2164-14-288 PMC3653781

[14] Raad G , Hazzouri M , Bottini S , Trabucchi M , Azoury J , Grandjean V . Paternal obesity: how bad is it for sperm quality and progeny health? Basic Clin Androl. 2017;27:20. 29123667 10.1186/s12610-017-0064-9 PMC5657098

[15] Shah DK , Missmer SA , Berry KF , Racowsky C , Ginsburg ES . Effect of obesity on oocyte and embryo quality in women undergoing in vitro fertilization. Obstet Gynecol. 2011;118(1):63‐70. 21691164 10.1097/AOG.0b013e31821fd360

[16] Steegers‐Theunissen RP , Verheijden‐Paulissen JJ , van Uitert EM , et al. Cohort profile: the Rotterdam periconceptional cohort (predict study). Int J Epidemiol. 2016;45(2):374‐381. 26224071 10.1093/ije/dyv147

[17] Heijnen EM , Eijkemans MJ , De Klerk C , et al. A mild treatment strategy for in‐vitro fertilisation: a randomised non‐inferiority trial. Lancet. 2007;369(9563):743‐749. 17336650 10.1016/S0140-6736(07)60360-2

[18] Hohmann FP , Macklon NS , Fauser BC . A randomized comparison of two ovarian stimulation protocols with gonadotropin‐releasing hormone (GnRH) antagonist cotreatment for in vitro fertilization commencing recombinant follicle‐stimulating hormone on cycle day 2 or 5 with the standard long GnRH agonist protocol. J Clin Endocrinol Metab. 2003;88(1):166‐173. 12519847 10.1210/jc.2002-020788

[19] Organization WH . WHO Laboratory Manual for the Examination and Processing of Human Semen. 5th ed. WHO; 2010.

[20] Ciray HN , Campbell A , Agerholm IE , Aguilar J , Chamayou S , Esbert M , et al. Proposed guidelines on the nomenclature and annotation of dynamic human embryo monitoring by a time‐lapse user group. Hum Reprod. 2014;29(12):2650‐2660. 25344070 10.1093/humrep/deu278

[21] Petersen BM , Boel M , Montag M , Gardner DK . Development of a generally applicable morphokinetic algorithm capable of predicting the implantation potential of embryos transferred on Day 3. Hum Reprod. 2016;31(10):2231‐2244. 27609980 10.1093/humrep/dew188 PMC5027927

[22] Prasad S , Tiwari M , Pandey AN , Shrivastav TG , Chaube SK . Impact of stress on oocyte quality and reproductive outcome. J Biomed Sci. 2016;23:36. 27026099 10.1186/s12929-016-0253-4 PMC4812655

[23] Bartolacci A , Buratini J , Moutier C , et al. Maternal body mass index affects embryo morphokinetics: a time‐lapse study. J Assist Reprod Genet. 2019;36(6):1109‐1116. 31062218 10.1007/s10815-019-01456-3 PMC6603074

[24] Bellver J , Mifsud A , Grau N , Privitera L , Meseguer M . Similar morphokinetic patterns in embryos derived from obese and normoweight infertile women: a time‐lapse study. Hum Reprod. 2013;28(3):794‐800. 23293223 10.1093/humrep/des438

[25] Leary C , Leese HJ , Sturmey RG . Human embryos from overweight and obese women display phenotypic and metabolic abnormalities. Hum Reprod. 2015;30(1):122‐132. 25391239 10.1093/humrep/deu276

[26] Bakos HW , Henshaw RC , Mitchell M , Lane M . Paternal body mass index is associated with decreased blastocyst development and reduced live birth rates following assisted reproductive technology. Fertil Steril. 2011;95(5):1700‐1704. 21145051 10.1016/j.fertnstert.2010.11.044

[27] Umul M , Kose SA , Bilen E , Altuncu AG , Oksay T , Guney M . Effect of increasing paternal body mass index on pregnancy and live birth rates in couples undergoing intracytoplasmic sperm injection. Andrologia. 2015;47(3):360‐364. 24717066 10.1111/and.12272

[28] Yang Q , Zhao F , Hu L , et al. Effect of paternal overweight or obesity on IVF treatment outcomes and the possible mechanisms involved. Sci Rep. 2016;6:29787. 27412918 10.1038/srep29787 PMC4944201

[29] Mushtaq R , Pundir J , Achilli C , Naji O , Khalaf Y , El‐Toukhy T . Effect of male body mass index on assisted reproduction treatment outcome: an updated systematic review and meta‐analysis. Reprod Biomed Online. 2018;36(4):459‐471. 29452915 10.1016/j.rbmo.2018.01.002

[30] Du Plessis SS, Cabler S , McAlister DA , Sabanegh E , Agarwal A . The effect of obesity on sperm disorders and male infertility. Nat Rev Urol. 2010;7(3):153‐161. 20157305 10.1038/nrurol.2010.6

[31] Tremellen K . Oxidative stress and male infertility—a clinical perspective. Hum Reprod Update. 2008;14(3):243‐258. 18281241 10.1093/humupd/dmn004

[32] Tesarik J . Paternal effects on cell division in the human preimplantation embryo. Reprod Biomed Online. 2005;10(3):370‐375. 15820045 10.1016/s1472-6483(10)61798-1

[33] Soubry A , Guo L , Huang Z , et al. Obesity‐related DNA methylation at imprinted genes in human sperm: results from the TIEGER study. Clin Epigenetics. 2016;8:51. 27158277 10.1186/s13148-016-0217-2 PMC4859994

[34] Oluwayiose OA , Wu H , Saddiki H , et al. Sperm DNA methylation mediates the association of male age on reproductive outcomes among couples undergoing infertility treatment. Sci Rep. 2021;11(1):3216. 33547328 10.1038/s41598-020-80857-2 PMC7864951

[35] Donkin I , Versteyhe S , Ingerslev LR , et al. Obesity and bariatric surgery drive epigenetic variation of spermatozoa in humans. Cell Metab. 2016;23(2):369‐378. 26669700 10.1016/j.cmet.2015.11.004

[36] Carone BR , Fauquier L , Habib N , et al. Paternally induced transgenerational environmental reprogramming of metabolic gene expression in mammals. Cell. 2010;143(7):1084‐1096. 21183072 10.1016/j.cell.2010.12.008 PMC3039484

[37] Fullston T , Palmer NO , Owens JA , Mitchell M , Bakos HW , Lane M . Diet‐induced paternal obesity in the absence of diabetes diminishes the reproductive health of two subsequent generations of mice. Hum Reprod. 2012;27(5):1391‐1400. 22357767 10.1093/humrep/des030

[38] Hoek J , Steegers‐Theunissen RPM , Willemsen SP , Schoenmakers S . Paternal folate status and sperm quality, pregnancy outcomes, and epigenetics: a systematic review and meta‐analysis. Mol Nutr Food Res. 2020;64(9):e1900696. 32032459 10.1002/mnfr.201900696 PMC7317557

[39] Pathak S , D'Souza R , Ankolkar M , Gaonkar R , Balasinor NH . Potential role of estrogen in regulation of the insulin‐like growth factor2‐H19 locus in the rat testis. Mol Cell Endocrinol. 2010;314(1):110‐117. 19683557 10.1016/j.mce.2009.08.005

[40] Dupont C , Kappeler L , Saget S , Grandjean V , Levy R . Role of miRNA in the transmission of metabolic diseases associated with paternal diet‐induced obesity. Front Genet. 2019;10:337. 31057600 10.3389/fgene.2019.00337 PMC6482346

[41] Swanson GM , Estill M , Diamond MP , et al. Human chromatin remodeler cofactor, RNA interactor, eraser and writer sperm RNAs responding to obesity. Epigenetics. 2020;15(1‐2):32‐46. 31354029 10.1080/15592294.2019.1644880 PMC6961666

[42] Sharma U , Rando OJ . Metabolic inputs into the epigenome. Cell Metab. 2017;25(3):544‐558. 28273477 10.1016/j.cmet.2017.02.003

[43] Guo L , Chao SB , Xiao L , et al. Sperm‐carried RNAs play critical roles in mouse embryonic development. Oncotarget. 2017;8(40):67394‐67405. 28978041 10.18632/oncotarget.18672 PMC5620181

[44] Yuan S , Schuster A , Tang C , et al. Sperm‐borne miRNAs and endo‐siRNAs are important for fertilization and preimplantation embryonic development. Development. 2016;143(4):635‐647. 26718009 10.1242/dev.131755 PMC4760322

