# Supplementary material for: A higher preconceptional paternal body mass index influences fertilization rate and preimplantation embryo development
Source: Andrology. 2021 Nov 25;10(3):486–94. doi: 10.1111/andr.13128 (PMC9299449; doi:10.1111/andr.13128)
Supplement: Supplementary file 1 — SUPPORTING INFORMATION [file ANDR-10-486-s002.docx]

**Supplemental table 1:** Baseline characteristics of the men in the study population of the Virtual Embryoscope cohort.

|  | **Normal weight**  **men** | **Overweight**  **men** | **Obese**  **men** | **p-value** |
| --- | --- | --- | --- | --- |
|  | **N=86** | **N=94** | **N=41** |  |
| **Age** (years) | 34.5 [31.8– 38.3] | 35.4 [32.1– 40.4] | 36.6 [30.9– 40.8] | **0.01** |
| **Geographic origin** |  |  |  | **0.01** |
| - Dutch | 76 (88.4%) | 73 (77.7%) | 30 (73.2%) |  |
| - Western | 3 (3.5%) | 2 (2.1%) | 1 (2.4%) |  |
| - Non-Western | 7 (8.1%) | 19 (20.2%) | 10 (24.4%) |  |
| **Educational level** |  |  |  | **0.01** |
| - Low | 12 (14.0%) | 17 (18.3%) | 9 (22.0%) |  |
| - Intermediate | 27 (31.4%) | 43 (46.2%) | 23 (56.1%) |  |
| - High | 47 (54.7%) | 33 (35.5%) | 9 (22.0%) |  |
| **BMI,** measured (kg/m^2^) | 23.2 [22.0-24.0] | 27.2 [26.1–28.1] | 32.2 [30.9-35.3] | **0.001** |
| **Waist-hip ratio** | 0.86 [0.82-0.89] | 0.89 [0.84-0.93] | 0.94 [0.90-0.97] | **0.001** |
| **Folic acid supplement use** |  |  |  | 0.20 |
| - 0,4-0,5 mg/day | 6 (7.0%) | 8 (8.5%) | 1 (2.4%) |  |
| - 5mg/day | 2 (2.3%) | 2 (2.1%) | 0 (0%) |  |
| **Vitamin use**, yes | 17 (22.7%) | 27 (32.5%) | 9 (23.7%) | 0.06 |
| **Alcohol consumption**, yes | 54 (62.8%) | 67 (71.3%) | 20 (48.8%) | **0.01** |
| **Smoking,** yes | 15 (17.4%) | 20 (21.3%) | 8 (19.5%) | 0.87 |

**Legend:** Data are presented as median [interquartile range (IQR)] or n (%).
