# Supplementary material for: A higher preconceptional paternal body mass index influences fertilization rate and preimplantation embryo development
Source: Andrology. 2021 Nov 25;10(3):486–94. doi: 10.1111/andr.13128 (PMC9299449; doi:10.1111/andr.13128)
Supplement: Supplementary file 2 — SUPPORTING INFORMATION [file ANDR-10-486-s001.docx]

**Supplemental table 2.** Fertilization and embryo usage rates in the total, IVF and ICSI study population of men stratified for categories of normal, overweight and obesity.

**Legend:** Data are presented as median [interquartile range (IQR)]. ^#^Significantly different (p<0.05) in comparison with normal weight men.

Abbreviations: IVF; in vitro fertilization, ICSI; intracytoplasmic sperm injection

| Treatment outcomes: | Normal weight men | | | Overweight men | | | Obese men | | |
| --- | --- | --- | --- | --- | --- | --- | --- | --- | --- |
|  | Total | IVF | ICSI | Total | IVF | ICSI | Total | IVF | ICSI |
| Fertilisation rate | 0.88  (0.74-1.00) | 0.89  (0.85-1.00) | 0.78  (0.67-1.00) | 0.81  (0.67-1.00) | 0.87  (0.80-1.00) | 0.72  (0.58-0.86) | 0.76^#^  (0.51-1.00) | 0.77  (0.46-1.00) | 0.72  (0.54-1.00) |
| Embryo usage rate | 0.67  (0.44 – 0.86) | 0.64  (0.42 – 0.84) | 0.67  (0.50 – 0.90) | 0.71  (0.50 - 1.00) | 0.73  (0.56 - 1.00) | 0.66  (0.50 –0.96) | 0.67  (0.50 -1.00) | 0.74  (0.50 - 1.00) | 0.65  (0.40 - 1.00) |
